# Supplementary material for: Exploring the effects of web-based psychological capitals training on teachers’ PsyCap development, emotional stability, and support: Evidence from Chinese inclusive education
Source: PLoS One. 2024 Sep 30;19(9):e0306453. doi: 10.1371/journal.pone.0306453 (PMC11441704; doi:10.1371/journal.pone.0306453)
Supplement: Attachment — Submitted filename: PONE-D-23-30803.pdf [file pone.0306453.s002.pdf]

PLOS ONE

Exploring the Effects of Web-based Psychological Capitals Training on Teachers' PsyCap Development, Emotional Stability, and Support: Evidence from Chinese Inclusive Education

--Manuscript Draft--

**Manuscript Number:**	PONE-D-23-30803
**Article Type:**	Research Article
**Full Title:**	Exploring the Effects of Web-based Psychological Capitals Training on Teachers' PsyCap Development, Emotional Stability, and Support: Evidence from Chinese Inclusive Education
**Short Title:**	Web-based Psychological Capitals Training
**Corresponding Author:**	YinRu SHI, Ph.D.<br>Lingnan Normal University<br>Zhanjiang, Guangdong CHINA
**Keywords:**	emotional stability; inclusive education; Intervention effect; psychological capitals (PsyCap); teacher support.
**Abstract:**	Inclusive education for students with autism spectrum disorders (ASD) has increasingly received attention nationwide in China. Schools realize that teachers are under stress and lack the skills to handle daily interactions with these students. So far, few studies have directed efforts to provide a remedy for teachers to improve their daily work. This study aimed to design and implement a 2-hour web-based training on psychological capital (PsyCap) to protect their well-being and foster their supportive behavior for ASD students in the inclusive class. A total of 120 targeted teachers were invited to participate in the training and were randomly divided into control and treatment groups. Pre-, post-, and follow-up surveys were distributed before, after, and one week after the training. ANOVA results suggested that teachers showed a significant increase in their PsyCap scores after completing the training, although the training effect slightly decreased after one week. Moreover, higher levels of PsyCap showed a positive influence on teachers' emotional stability and supportive behavior in class. Our findings highlighted the importance of nurturing teachers' PsyCap to combat stress and provide more support for ASD students on a daily basis.
**Order of Authors:**	YinRu SHI, Ph.D.
	Kuen Fung Kenneth SIN, Ph.D.
**Additional Information:**	
**Question**	**Response**
**Financial Disclosure**<br><br>Enter a financial disclosure statement that describes the sources of funding for the work included in this submission. Review the submission guidelines for detailed requirements. View published research articles from *PLOS ONE* for specific examples.<br><br>This statement is required for submission and **will appear in the published article** if the submission is accepted. Please make sure it is accurate.	Yes

Powered by Editorial Manager® and ProduXion Manager® from Aries Systems Corporation

**Funded studies**<br>Enter a statement with the following details:<br>• Initials of the authors who received each award<br>• Grant numbers awarded to each author<br>• The full name of each funder<br>• URL of each funder website<br>• Did the sponsors or funders play any role in the study design, data collection and analysis, decision to publish, or preparation of the manuscript?<br><br>Did you receive funding for this work?	
Please add funding details.<br>as follow-up to "**Financial Disclosure**<br><br>Enter a financial disclosure statement that describes the sources of funding for the work included in this submission. Review the submission guidelines for detailed requirements. View published research articles from *PLOS ONE* for specific examples.<br><br>This statement is required for submission and **will appear in the published article** if the submission is accepted. Please make sure it is accurate.<br><br>**Funded studies**<br>Enter a statement with the following details:<br>• Initials of the authors who received each award<br>• Grant numbers awarded to each author<br>• The full name of each funder<br>• URL of each funder website<br>• Did the sponsors or funders play any role in the study design, data collection and analysis, decision to publish, or preparation of the manuscript?<br><br>Did you receive funding for this work?"	The authors would like to acknowledge the funding support provided by the Department of Education of Guangdong Province, China, through the Special Innovation Project for Guangdong Provincial Higher Education Institutions in 2022. The project number is 2022WQNCX042.
Please select the country of your main research funder (please select carefully as in some cases this is used in fee calculation).	CHINA - CN

as follow-up to "**Financial Disclosure**<br><br>Enter a financial disclosure statement that describes the sources of funding for the work included in this submission. Review the submission guidelines for detailed requirements. View published research articles from *PLOS ONE* for specific examples.<br><br>This statement is required for submission and **will appear in the published article** if the submission is accepted. Please make sure it is accurate.<br><br>**Funded studies**<br>Enter a statement with the following details:<br>• Initials of the authors who received each award<br>• Grant numbers awarded to each author<br>• The full name of each funder<br>• URL of each funder website<br>• Did the sponsors or funders play any role in the study design, data collection and analysis, decision to publish, or preparation of the manuscript?<br><br>Did you receive funding for this work?"	
**Competing Interests**<br><br>Use the instructions below to enter a competing interest statement for this submission. On behalf of all authors, disclose any competing interests that could be perceived to bias this work—acknowledging all financial support and any other relevant financial or non-financial competing interests.<br><br>This statement is required for submission and **will appear in the published article** if the submission is accepted. Please make sure it is accurate and that any funding sources listed in your Funding Information later in the submission form are also declared in your Financial Disclosure statement.	The authors have declared that no competing interests exist.

View published research articles from *PLOS ONE* for specific examples.<br><br>**NO authors have competing interests**<br><br>Enter: *The authors have declared that no competing interests exist.*<br><br>**Authors with competing interests**<br><br>Enter competing interest details beginning with this statement:<br><br>*I have read the journal's policy and the authors of this manuscript have the following competing interests: [insert competing interests here]*<br><br>* typeset	
**Ethics Statement**<br><br>Enter an ethics statement for this submission. This statement is required if the study involved:<br><br>• Human participants<br>• Human specimens or tissue<br>• Vertebrate animals or cephalopods<br>• Vertebrate embryos or tissues<br>• Field research<br><br>Write "N/A" if the submission does not require an ethics statement.<br><br>General guidance is provided below. Consult the submission guidelines for detailed instructions. **Make sure that all information entered here is included in the Methods section of the manuscript.**	The study involving human participants was reviewed and approved by the Department of Special Education and Counselling at The Education University of Hong Kong. Written consent was obtained from the study participants included in the study.

**Format for specific study types**<br><br>**Human Subject Research (involving human participants and/or tissue)**<br>• Give the name of the institutional review board or ethics committee that approved the study<br>• Include the approval number and/or a statement indicating approval of this research<br>• Indicate the form of consent obtained (written/oral) or the reason that consent was not obtained (e.g. the data were analyzed anonymously)<br><br>**Animal Research (involving vertebrate animals, embryos or tissues)**<br>• Provide the name of the Institutional Animal Care and Use Committee (IACUC) or other relevant ethics board that reviewed the study protocol, and indicate whether they approved this research or granted a formal waiver of ethical approval<br>• Include an approval number if one was obtained<br>• If the study involved *non-human primates*, add *additional details* about animal welfare and steps taken to ameliorate suffering<br>• If anesthesia, euthanasia, or any kind of animal sacrifice is part of the study, include briefly which substances and/or methods were applied<br><br>**Field Research**<br><br>Include the following details if this study involves the collection of plant, animal, or other materials from a natural setting:<br>• Field permit number<br>• Name of the institution or relevant body that granted permission	
**Data Availability**<br><br>Authors are required to make all data underlying the findings described fully available, without restriction, and from the time of publication. PLOS allows rare exceptions to address legal and ethical concerns. See the PLOS Data Policy and FAQ for detailed information.	Yes - all data are fully available without restriction

A Data Availability Statement describing where the data can be found is required at submission. Your answers to this question constitute the Data Availability Statement and **will be published in the article**, if accepted.<br><br>**Important:** Stating 'data available on request from the author' is not sufficient. If your data are only available upon request, select 'No' for the first question and explain your exceptional situation in the text box.<br><br>Do the authors confirm that all data underlying the findings described in their manuscript are fully available without restriction?	
**Describe where the data may be found in full sentences. If you are copying our sample text, replace any instances of XXX with the appropriate details.**<br><br>• If the data are **held or will be held in a public repository**, include URLs, accession numbers or DOIs. If this information will only be available after acceptance, indicate this by ticking the box below. For example: *All XXX files are available from the XXX database (accession number(s) XXX, XXX.*).<br>• If the data are all contained **within the manuscript and/or Supporting Information files**, enter the following: *All relevant data are within the manuscript and its Supporting Information files.*<br>• If neither of these applies but you are able to provide **details of access elsewhere**, with or without limitations, please do so. For example:<br><br>*Data cannot be shared publicly because of [XXX]. Data are available from the XXX Institutional Data Access / Ethics Committee (contact via XXX) for researchers who meet the criteria for access to confidential data.*<br><br>*The data underlying the results presented in the study are available from (include the name of the third party*	The data underlying the results presented in the study are available from the first author upon request.

Powered by Editorial Manager® and ProduXion Manager® from Aries Systems Corporation

and contact information or URL).<br>• This text is appropriate if the data are owned by a third party and authors do not have permission to share the data.<br><br>* typeset	
Additional data availability information:	Tick here if the URLs/accession numbers/DOIs will be available only after acceptance of the manuscript for publication so that we can ensure their inclusion before publication.

Manuscript
Click here to access/download;Manuscript;ManuscriptNewUpdated.docx

Exploring the Effects of Web-based Psychological Capitals Training on Teachers' PsyCap Development, Emotional Stability, and Support: Evidence from Chinese Inclusive Education

Yin Ru SHI* and Kuen Fung Kenneth SIN

Dr. Yin Ru **SHI** (corresponding author)
Shiyinru921020@163.com
Department of Special Education, Lingnan Normal University, Zhanjiang, China.
29 Cunjin Road, Chikan, Zhanjiang, Guangdong, China

Prof. Dr. Kuen Fung Kenneth **SIN**
kfsin@eduhk.hk
Department of Special Education and Counselling, The Education University of Hong Kong, Hong Kong, China

Abstract

Inclusive education for students with autism spectrum disorders (ASD) has increasingly received attention nationwide in China. Schools realize that teachers are under stress and lack the skills to handle daily interactions with these students. So far, few studies have directed efforts to provide a remedy for teachers to improve their daily work. This study aimed to design and implement a 2-hour web-based training on psychological capital (PsyCap) to protect their well-being and foster their supportive behavior for ASD students in the inclusive class. A total of 120 targeted teachers were invited to participate in the training and were randomly divided into control and treatment groups. Pre-, post-, and follow-up surveys were distributed before, after, and one week after the training. ANOVA results suggested that teachers showed a significant increase in their PsyCap scores after completing the training, although the training effect slightly decreased after one week. Moreover, higher levels of PsyCap showed a positive influence on teachers' emotional stability and supportive behavior in class. Our findings highlighted the importance of nurturing teachers' PsyCap to combat stress and provide more support for ASD students on a daily basis.

Keywords: emotional stability, inclusive education, intervention effect, psychological capitals (PsyCap), teacher support.

Web-based Psychological Capitals Training

Inclusive education receives a growing concern from international communities as increasingly more educational reforms have taken place in both developing and developed regions for a heightened understanding of education as a human right (1). However, inclusive education is challenging to implement (2). It attempts to accommodate students with various developmental problems from different cultural and socioeconomic backgrounds, and family structures. Among the children with special needs, children with autism spectrum disorders (ASD) are a group of minorities that receive great attention from researchers and educators. These children show the complexity of everyday needs (3). Teachers thus play a crucial role because they are in charge of implementing and facilitating inclusive practice with all children in the class (4). To successfully implement inclusive education practice, teachers must develop a strong understanding of domain knowledge and the necessary skills for addressing classroom challenges and a positive mindset that is unprejudiced toward students with special needs (5). However, Tristani and Bassett- Gunter (6) pointed out that teachers are inadequately prepared to teach children with ASD in inclusive settings. Schools, with radical inclusive educational reforms, face difficulties, changes, growing standards, and accountability, putting pressure on their teachers (7). In addition, student misconduct and growing workload for tailor-made care to students adds extra burden on teachers' shoulders with the reforms (8). These factors generate stress, pessimism, and a lack of motivation in teachers in general, which may have a detrimental impact on their job motivation and performance.

Unfortunately, these stress factors are beyond teachers' control. However, Forcina (9) pointed out that "hope for stress moderation lies within the individual teachers and their ability to manage the social and emotional demands of the profession"(p.6). Researchers therefore claimed that an individual in the future needs a new kind of capital, namely,

Psychological Capitals (PsyCap, 10) to develop their ability to cope with hardships and crises. According to Peterson et al. (11), PsyCap is a critical resource for motivation of success, cognitive functioning, and individual performance, including four malleable and manageable constructs: hope, optimism, resilience, and self-efficacy (12). In their meta-analysis study, Avey and colleagues (13) concluded a significant negative relationship between PsyCap and undesirable employee attitudes (e.g., cynicism, turnover intentions, job stress, and anxiety) and undesirable employee behavior (e.g., deviance). Additionally, the authors also found positive relationships between PsyCap and desirable employee attitudes (e.g., well-being, satisfaction, commitment) and behavior (e.g., helping, support). PsyCap research in teacher samples also evidenced that PsyCap can buffer effectively when teachers' emotional demands are high at workplace (14). In addition, previous results supported that teachers with PsyCap tend to be more resourceful to engage in supportive behavior in class (15).

The motivation behind this study is derived from the fact that PsyCap research was very limited in inclusive education contexts. In fact, teachers face a growing number of challenges brought up by inclusive education reforms, and they find themselves lacking adequate preparation to involve ASD students in daily teaching and learning. Therefore, professional development in psychological resources seemed to be particularly crucial for teachers to tackle challenges more effectively in inclusive classrooms. PsyCap may assist schools in dealing with obstacles and revitalizing the inner resources of these teachers via positive psychology-based training programs. Past studies evidenced that PsyCap Training can be an effective way to develop and enhance the positive psychological resources of self-efficacy, optimism, hope, and resilience, leading to benefits such as increased job satisfaction, performance, and well-being. In past investigations, Luthans and his associates

Web-based Psychological Capitals Training

(16, 17) showed that it is possible to develop PsyCap through brief training interventions, which could potentially involve the utilization of technology-facilitated delivery methods. For example, Luthans et al. (17) reported on an experimental study that examined the effectiveness of an intensive 2-hour web-based training intervention to develop positive PsyCap in university students. The intervention involved a series of online modules that aimed to enhance students' self-efficacy, optimism, hope, and resilience. Results showed that students who received the web-based training had significant improvements in their levels of self-efficacy, optimism, and hope compared to a control group. In this study, an online PsyCap training was designed based on the design of Luthans et al. (17), aiming to provide effective buffer for inclusive education teachers to tackle with negative stressors and to provide timely support to the students with special needs. This study aimed to answer the following research questions:

RQ1: Can teachers' PsyCap be effectively developed through the short web-based PsyCap training?

RQ2: Can this PsyCap training show sustained positive effects on teachers' PsyCap to be developed?

RQ3: How effective can this PsyCap training on teachers' sustained well-being and support to students?

Literature Review

Theoretical Background

In China, the concept of inclusive education emerged in the late 1980s, and since then, it has gained increasing attention from policymakers and educators. In 2010, the Chinese government launched the "Action Plan for the Development of Special Education (2010-2020)," which aimed to promote inclusive education and improve the quality of special

education (18). In response to the call for the equal right to education, the Chinese government has set a primary goal to increase school enrolment and retention by integrating children with ASD into mainstream schools to achieve the universalization of compulsory education (19). Despite government efforts, challenges persist. Insufficient resources, including a lack of assistive technology, adapted materials, and appropriate facilities, pose a challenge for learners with diverse needs (20). Furthermore, a lack of awareness and understanding of inclusive education among the general public contributes to stigma and discrimination towards these learners. However, the primary challenge lies in the inadequate teacher training on inclusive education. Many Chinese teachers lack the necessary knowledge and skills to effectively teach diverse learners (18).

Inclusive education, which aims to ensure that all students have access to quality education regardless of their background, has put increasingly more stress on teachers' shoulders nowadays. In China, such concern is also highlighted by educators and school leaders (21). Teachers reported heightened stress when implementing inclusive practice in regular classrooms. For example, Brackenreed (22) identified the most common stressors during inclusion, such as workload, time management, lack of general support and locus of control, and insufficient teacher preparation. In addition, Jennett et al. (23) warned that teachers might be especially at risk for burnout in inclusive education when they work with ASD students. Taking into account the meta-analysis of Aloe et al. (24), behavioral disturbances in day-to-day interaction with students result in teachers' emotional exhaustion. Feelings of helplessness and loss of control also exacerbate stress. Increasingly more researchers advocated that teachers in such situations should be given more opportunities to cultivate positive organizational behavior (POB). Following this, Luthans (25) introduced POB as a means to apply positive psychology in the workplace. The call for a positive

approach to psychology is not a new concept. Maslow (26) suggested that psychology was too focused on negative aspects and should balance it with positive constructs. Positive psychology emerged in the literature in 2000, introducing positive constructs such as hope, optimism, subjective well-being, and individual development (27). POB is defined as positively oriented human resource strengths and psychological capacities that can be measured, developed, and managed for performance improvement in today's workplace (25). It emphasizes positive constructs that are state-like and thus open to development. Thus, organizations can benefit from cultivating and leveraging the positive psychological resources of their employees, leading to improved performance and well-being. Research has shown that promoting POB among teachers leads to increased job performance and satisfaction, reduced stress and burnout, and improved student outcomes (28). Furthermore, positive attitudes and behaviors among teachers have been found to be contagious and can positively impact students' attitudes and behaviors (29). Thus, it is crucial for schools to put more effort in cultivating POB among their teachers to create a positive and inclusive learning environment. To gain a comprehensive understanding of psychological resources in organizational settings, it is crucial to examine the theoretical foundations of POB and its specific construct of PsyCap. PsyCap is a psychological resource that has been mentioned in various fields, including economics, sociology, and organizational psychology, and is believed to promote personal growth and well-being, which in turn can lead to organizational growth (10, 30). Researchers have identified PsyCap as a valuable resource for protecting individuals from work-related stress (12). Individuals with PsyCap are thought to be more equipped to adapt to and cope with stressful events, which can result in greater emotional stability, optimism, and cheerfulness in the face of negative stressors at work (31). These teachers are likely to be psychologically resourceful, demonstrating qualities such as hope,

optimism, resilience, and efficacy, which enable them to adapt to changing demands and evaluate problems in a balanced way. Existing studies on teachers with high levels of PsyCap have demonstrated promising results, such as high levels of work engagement, well-being, and interpersonal harmony (32). PsyCap may be particularly crucial for teachers in demanding situations. Research has consistently shown that teachers with higher levels of PsyCap tend to exhibit more supportive behaviors towards their students (13). PsyCap enhances the teacher's ability to cope with stress, develop positive relationships with students, and promote student motivation and engagement (32). Specifically, teachers with higher levels of PsyCap tend to have a greater sense of self-efficacy, which leads to a more proactive and goal-oriented approach to teaching, using creative and innovative teaching strategies to meet the diverse needs of their students (32). Similarly, teachers with higher levels of PsyCap tend to show optimism and hope, contributing to more teacher support, because they are better able to manage stress and job demands, and maintain a positive and supportive attitude towards their students (33). Therefore, empowering teachers with PsyCap can promote teachers' well-being and foster their support for better students' adaptation and performance in class.

PsyCap with Four Components

PsyCap encompasses four components: self-efficacy, optimism, hope, and resilience. These components have been extensively studied in the literature and have significant implications for individuals' well-being and performance in educational settings (12). Hope, as described by Luthans and Youssef (12), involves perceiving a goal, identifying pathways to achieve it, and being motivated to pursue it. It plays a crucial role in educational contexts by providing individuals with a sense of direction and purpose. Research has shown that higher levels of hope are associated with greater academic motivation, engagement, and

achievement (34). Students with high levels of hope are more likely to set challenging goals, develop effective strategies, and persist in the face of obstacles. Self-efficacy, another component of PsyCap, refers to an individual's belief in their ability to succeed in specific tasks or situations. This belief influences individuals' choices, effort, and persistence in academic endeavors. Students with high self-efficacy are more likely to set challenging goals, exert greater effort, and demonstrate higher levels of academic performance (35). Developing self-efficacy can be facilitated through various strategies, including mastery experiences, social modeling, and verbal persuasion. Providing students with opportunities to successfully accomplish tasks, exposing them to positive role models, and offering supportive feedback can contribute to the development of self-efficacy beliefs. Resilience, the ability to bounce back from setbacks and difficulties, is another vital component of PsyCap in educational contexts. Students often face academic challenges, such as exams, assignments, and academic stress, which can impact their well-being and academic performance. Resilience helps students effectively cope with adversity and maintain their psychological well-being. Research has shown that resilient individuals exhibit higher levels of psychological well-being, adaptive coping strategies, and academic success (36). Developing resilience can be supported through interventions focused on stress management, emotion regulation, and coping skills training. Optimism, the fourth component of PsyCap, involves the expectation of positive outcomes. Optimistic individuals tend to have a positive outlook on the future, viewing challenges as opportunities for growth and improvement. In educational settings, optimism has been linked to higher levels of academic motivation, engagement, and achievement (37). Cognitive restructuring, a technique that involves challenging negative thoughts and replacing them with positive and realistic ones, and positive self-talk are effective strategies to develop and enhance optimism.

Web-based Psychological Capitals Training

It is important to note that PsyCap is not a fixed trait but can be developed and strengthened through training interventions. PsyCap training provides individuals with a range of tools and techniques to enhance their positive psychological resources, ultimately contributing to their success in educational settings. By focusing on the development of self-efficacy, hope, resilience, and optimism, individuals can acquire the necessary skills and mindset to navigate challenges, overcome obstacles, and thrive academically.

Emotional Wellbeing

Teachers play a crucial role in ensuring the success of inclusive education, as they are responsible for creating a supportive and inclusive learning environment that meets the needs of diverse learners. However, the demands and challenges of inclusive education can affect teachers' emotional wellbeing, which can have negative consequences for both teachers and students. Inclusive education is associated with increased demands on teachers, such as managing diverse classrooms, adapting instruction to meet individual students' needs, and collaborating with other professionals and parents (38). These demands can lead to emotional exhaustion, stress, and burnout, which can negatively impact teachers' wellbeing (39). Research has shown that teachers who experience emotional exhaustion and burnout are more likely to report lower job satisfaction, decreased commitment to teaching, and higher turnover rates (40). In addition, teachers' emotional wellbeing can also impact student outcomes. A study by Jia et al. (41) found that teachers' emotional exhaustion negatively impacted student achievement, while teachers' emotional intelligence positively predicted academic performance. In addition, teachers' job satisfaction was positively associated with students' academic achievement and motivation. To support teachers' emotional wellbeing in inclusive education settings, various strategies have been proposed in the literature. For example, self-care strategies, such as mindfulness practices and physical exercise, can help teachers manage

their stress and improve their emotional wellbeing (40). In addition, providing teachers with professional development opportunities to enhance their knowledge and skills in inclusive education can be a good means to improving their confidence and reducing their stress (39).

Teacher Support

In inclusive education, teachers are required to provide social support to students to meet their unique needs. According to Anderson et al. (42), social support is essential for successful inclusive education. Social support is a multifaceted construct that refers to the provision of emotional, instrumental, informational, and appraisal assistance from others. Emotional support involves providing comfort, empathy, and understanding, while instrumental support involves providing practical assistance, such as transportation, financial support, or physical care. Informational support involves providing guidance, advice, or information, while appraisal support involves providing feedback, encouragement, and affirmation (43). Teachers who provide social support can help students with special needs feel valued, respected, and included in the classroom, which can improve their academic and social outcomes. One study conducted by Tzuriel and Shamir (44) found that social support from teachers was positively related to students' academic achievement and social integration in inclusive education settings. Additionally, other studies have found that teachers who provide social support can reduce students' anxiety and stress levels and increase their self-esteem and self-efficacy (42). However, providing social support in inclusive education can be challenging for teachers. According to Lopata et al. (45), teachers in inclusive education settings have reported that they are overwhelmed, stressed, and underprepared to meet the unique needs of their students. They also reported the lack of training in necessary knowledge and skills to provide social support in inclusive class.

Web-based PsyCap Training Design

Web-based Psychological Capitals Training

Insufficient teacher training in inclusive context becomes the critical hurdle for teachers to carry out classroom practice smoothly (46, 47). Teachers may have limited knowledge about how to accommodate the unique characteristics of children with ASD to pedagogical adaptations (23). For example, teachers who lack expertise show a hard time applying specialist knowledge, tailoring students' special needs, and coping with stressful events in inclusive classrooms (6). To better provide equal educational opportunities for all learners, including those with diverse needs, well-trained and competent teachers are urgently needed. Thus, professional development is desirable as it offers a constructive, systematic approach to supporting teachers in fostering a sense of efficacy and competence by providing them with the necessary knowledge and skills to teach diverse learners effectively (48).

Teachers' professional development on PsyCap typically involves a series of workshops or sessions that focus on building individuals' positive psychological resources, such as self-efficacy, optimism, hope, and resilience. The design of PsyCap Training varies depending on the specific program, but most programs follow the design framework. (1) Assessment: before starting the training, participants may complete assessments of their current levels of PsyCap. (2) Education: participants learn about the concepts of self-efficacy, optimism, hope, and resilience, including the benefits of each for personal and professional development. (3) Skill-building: participants engage in activities and exercises designed to develop and enhance their PsyCap. These may include goal-setting, cognitive restructuring, visualization techniques, and stress management strategies. (4) Reinforcement: positive feedback and reinforcement are used to reinforce positive behaviors and attitudes. (5) Maintenance: follow-up sessions or activities may be provided to help participants maintain the gains made during the training.

Web-based Psychological Capitals Training

Past studies suggested that the use of online intervention technology has several advantages over traditional face-to-face interventions. PsyCap training programs have also been modified with technology-based features to accommodate the demands for online training (49, 50). Luthans et al. (17) supported the use of the online PsyCap training intervention as their meta-analytic results indicated that web-based instruction might be as effective, or even more effective than traditional face-to-face classroom instruction. Online interventions are easily accessible to a large number of people, can be completed at any time or place, and are often more cost-effective than in-person interventions. In addition, online interventions may be particularly beneficial for individuals who are hesitant to seek help due to social stigma or other barriers. Online training with technology supports such as personalized animation, detailed PowerPoints, personalized exercises, and video commentary by a facilitator can maximize the learning and development of PsyCap (17).

Web-based interventions consisting of multiple modules aimed at developing self-efficacy, hope, resilience, and optimism, have been used to deliver PsyCap Training. For example, the study by Sumalrot et al. (50) aimed to evaluate the effectiveness of a web-based PsyCap intervention on the mental well-being of tourism workers during the COVID-19 pandemic. The intervention was designed to enhance self-efficacy, hope, and resilience over a 6-week period through various online activities. A randomized controlled trial was conducted, and the intervention group demonstrated significant improvements in their overall mental health and well-being compared to the control group. The study implied that the web-based delivery of the PsyCap intervention provided a flexible and accessible approach that can be adapted to various contexts. Therefore, organizations can implement similar interventions to support the mental health of their employees. Overall, the existing literature suggested that online PsyCap Training can be an effective way to develop and enhance the positive

psychological resources of self-efficacy, optimism, hope, and resilience, leading to benefits such as increased job satisfaction, performance, well-being (10, 13) and reducing symptoms of depression and anxiety (51).

Method

Sample Data Collection

This study used a pre- and post-test quasi-experimental design using a sample of 120 primary school teachers teaching inclusive classes in a coastal city of China. Recruitment of the target teachers started on April 20th, 2020. The sample size for the treatment group included 60 target teachers, and the control group contained 60 teachers. The teachers were randomly assigned into the two groups. Teachers in the treatment group were provided with the online PsyCap Training, while teachers in the control group were not given the training, but had a decision-making exercise instead. We developed the teacher pre- and post-surveys online using a Chinese data collection platform which has access to inclusive teacher samples in China. This study involved target teachers and asked them to create their own unique ID with a combination of their surname and the last four digits of their phone number, such that we could match their pre- and post-surveys after submission. These teachers completed the pre-surveys on their demographic information and baseline PsyCap (T1) one week before they were randomly assigned to either a 2-hour PsyCap teacher training (treatment group N = 60) or a decision-making exercise that included the same time duration and multimedia techniques (control group N= 60). Both treatment and control groups received a post-survey on PsyCap, emotional stability, and teacher support upon completion of the intervention (T2). After a week, the teachers in the treatment group were further invited for a follow-up survey (T3) on these key variables.

Web-based Psychological Capitals Training

Target teachers were contacted through personal email for participation consent in our online "Positive Quality Teacher Training" project. In this invitation, the research topic and purpose were explained in detail. Written consent was obtained from the teachers.

In total, 178 teachers were invited, and 120 of them agreed to participate in our study and included for data analysis (response rate = .67). Most of the teachers were female (90.1%). Their age ranged from 21 to 50, with 47.6% between 21 and 30, 32.2% between 31 and 40, and 2.1 percent missing. Most of them (84.7%) received a university education. Their work tenure ranged from 1 year to 30 years. The average age of the students they taught was 8.6 years old (SD = 2.31).

Intervention Procedures

Based on the web-based PsyCap training design proposed by Luthans et al. (17), our study also emphasized the effectiveness of personalized animation, detailed PowerPoints, personalized exercises, and video commentary by a facilitator in enhancing learning and development of PsyCap. The personalized animation and video commentary provide visual aids that can help the learners better understand the concepts and make the learning process more engaging. The detailed PowerPoints provide a structure for the learners to follow and provide clear and concise information on the topic being covered. The personalized exercises provide a practical application of the concepts, which can help learners to internalize and retain the information.

In this study, the teachers were provided a 2-hour web-based training session on PsyCap. The implementation of the intervention for the treatment group included two online sessions. The first session included a one-hour narrated interactive PowerPoint presentation on PsyCap training embedded in flash animation, with short video clips from popular movies used as examples of self-efficacy, hope, optimism, and resilience in dramatized settings. The

second session included 50 minutes of personalized exercises in developing self-efficacy, hope, optimism, and resilience. In the second session, for the development of the four components of PsyCap, the teachers were asked to write an interactive teaching plan for their inclusive students for the coming class. They were asked to write down the importance of personal values and setting realistically challenging and personally valuable goals to complete the teaching plan. The teachers were directed to take the goals that were realistically challenging and break them down into smaller goals. This process of dividing large goals into smaller more manageable ones was also designed to increase the ability of hope. For the development of self-efficacy, Bandura (35) suggested that mastery experiences can help build self-efficacy. Mastery experiences are based on direct, personal experience rather than secondhand accounts, which allow us to observe direct links between effort and performance, thereby increasing expectancy judgments about our ability to perform well in particular situations. Previously, the teachers were asked to start with small challenges and gradually increase the difficulty level during the teaching plan design. They were also asked to recall other successful colleagues who had done similar tasks, and how their colleagues feel and react when receiving constructive feedback from their students. Moreover, the teachers were also taught the tactic of self-persuasion, such that they could provide positive feedback and encouragement to motivate themselves to exert greater effort and ability to achieve their teaching plans in the inclusive class. The increased positive expectations about those outcomes were intended to contribute to developing optimism for achieving success. In addition, when the teachers practiced developing strategies to attain personal goals, negative expectations may be reduced, and thus, positively influence optimism (51). Moreover, while the teachers were doing the teaching plan task, they were taught the importance of developing resilience of PsyCap. Particularly, they were introduced the tactics such as facing reality,

creating meaning in life, and improvising to find new ways to reach goals in building resilience to build up a better basis for enduring hardship.

Measures

Psychological Capitals. We adopted a 12-item scale short version of PsyCap questionnaire (17) from the original 24-item PsyCap questionnaire (52). In our study, the 12-item short questionnaire included 3 items for self-efficacy, hope, optimism, and resilience respectively. To emphasize the "state-like" nature of the measure, the participants were asked to respond by describing "how you may think about yourself right now." Each PsyCap component demonstrated acceptable reliability in our study from T1 to T3 (efficacy =.871-.890, hope = .761-.825, resilience = .865-.901, optimism = .760-.804), as well as overall PsyCap (.923-.943). Although psychometric support for the construct validity of PsyCap was demonstrated in many past PsyCap research (see 51), limited evidence of validity was reported in the inclusive education context. Therefore, confirmatory factor analysis of PsyCap was conducted in our study while considering PsyCap as a second-order factor (see theoretical discussion of second-order structure in Luthans et al., (17)). All of the item loadings were significant ($p < .01$) on their respective latent factor as well as each component loading on the second-order factor PsyCap. Results of the CFA were as follows: CFI =.920-.958, TLI = .926-.945, RMSEA = .050-.054. CFA results suggested good fit for the second-order factor model of PsyCap. Unless otherwise mentioned, all scales used in our study were anchored on a 5-point Likert scale ranging from 1 (strongly disagree) to 5 (strongly agree).

Teacher Support. Based on the original 60-item child and adolescent social support scale (CASSS) developed by Malecki et al. (53), we made further modifications to create a shortened version consisting of 10 items. This modified scale aimed to assess teachers'

support in inclusive education. The development of the shortened scale was based on existing research on the CASSS scale and its psychometric properties. By selecting a subset of the original 60 items from the CASSS scale that were most representative of teachers' support, we aimed to capture the essential aspects of this construct while reducing respondent fatigue and increasing response rates. The selection of items was guided by theoretical considerations and expert judgment in consultation with educational researchers in the inclusive education field. In our study, the internal consistency reliability of the 10-item scale was evaluated using Cronbach’s alpha, which was calculated to be .961-.977 (T2 and T3). This high value suggests that the items in the scale were highly interrelated and exhibited strong internal consistency, indicating that the scale was reliable for measuring teachers’ support in the inclusive education context.

Emotional Stability. Chaturvedi and Chander (31) developed a 16-item emotional stability scale to measure individuals' emotional stability. The development of this scale was guided by existing theories and research on personality traits, emotional well-being, and psychological resilience to capture the multifaceted nature of emotional stability. The scale included items that assessed emotional regulation, self-control, resilience, and adaptability. These dimensions reflect the underlying constructs of emotional stability, which have been consistently linked to individuals' overall psychological well-being and positive life outcomes (54). In our study, Cronbach's alpha coefficient was .861-.920 (T2 and T3), indicating strong internal consistency.

Results

Descriptive analysis

Table 1 shows the descriptive statistics, reliabilities, and Pearson's correlations of the key study variables using the post-survey sample. Results showed that PsyCap, emotional

stability, and teacher support were positively and significantly correlated with each other. Data were checked for normality and outliners. A few cases of extreme outliers were treated as missing (e.g., age = 120).

Insert Table 1 Here

Effectiveness of the PsyCap training

To answer **Q1**, we investigated how this 2-hour web-based PsyCap training could effectively influence students' development of PsyCap using data from T1 and T2. Firstly, we conducted an independent sample T test for treatment and control groups to make sure that the teachers' baseline PsyCap (T1) was roughly similar across the groups. Results showed no significant differences of PsyCap between the control group (M = 40.933, SD = 3.755) and the treatment group (M = 40.917, SD = 3.369), t(118) = .026, p = .979. Then, we conducted mixed ANOVA. Specifically, we aimed to explore the effect of PsyCap training with a 2 (condition: treatment vs. control) x 2 (time: pre-test vs. post-test) factorial quasi-experimental design among 120 participating teachers. The Shapiro-Wilk test indicated that the data were normally distributed, W = 0.92, p = 0.176. The Levene's test indicated that the assumption of homogeneity of variances was met, F(1, 118) = 2.337, p = .129. The results revealed a significant main effect of condition, F(1, 118) = 90.688, p < .001, η^2 = .435, indicating that the treatment group (M = 57.617, SD = 2.337) had significantly higher PsyCap scores than the control group (M = 45.167, SD = 5.944). There was also a significant main effect of Time, F(1, 118) = 910.711, p < .001, η^2 = .885, indicating that teachers' PsyCap significantly increased from the pre-test (M = 40.925, SD = 3.505) to the post-test (M = 51.392, SD = 7.701) regardless of treatment condition. Additionally, the results also indicated a significant

interaction effect (Figure 1) between condition and time, $F(1, 118) = 323.001$, $p < .001$, $\eta^2 = .732$. All the effect sizes for the main effects of condition, time, and their interaction were large ($\eta^2 = .435$, $\eta^2 = .885$, and $\eta^2 = .732$, respectively). Moreover, paired sample T tests were conducted for both control and treatment groups respectively. Results indeed showed a significant increase in PsyCap for both control($t(59) = -7.368$, $p < .001$) and treatment ($t(59) = -42.974$, $p < .001$) groups over time regardless treatment condition. Nevertheless, treatment group (Δdiff = 16.700) showed much significant increase in PsyCap than the control group (Δdiff = 4.233).

Insert Figure 1 Here

We also conducted mixed ANOVA for each specific components of PsyCap (i.e., self-efficacy, hope, resilience, and optimism) in order to further examine in-depth how this PsyCap training could nurture the four components of PsyCap. Results showed similar patterns, indicating significant main effects of condition and time, and significant interaction effects of condition x time on the four components of PsyCap, namely self-efficacy, hope, resilience, and optimism. The online PsyCap training seemed to have large effect sizes on the development of teachers' PsyCap, as well as its four components. Figure 2 shows the interaction plots for the four components of PsyCap.

Insert Figure 2 Here

Sustainability of the PsyCap Training

Web-based Psychological Capitals Training

In **Q1**, we predominantly investigated the effect of the PsyCap training on teachers' development of PsyCap during the training. For **Q2**, we focused on investigating the sustainability of the training effect after completion of the training. Table 2 shows the Pearson's correlations of teachers' PsyCap from pre-test (T1), post-test(T2), and follow-up surveys (T3). One-way repeated measures ANOVA was conducted to investigate the impact of the web-based training on teachers' PsyCap before the training (pre-test), immediately after the training (post-test), and one week following the training (follow-up). Mauchly's test indicated that the assumption of sphericity had been violated, $\chi^2(2) = .507$, $p < .001$. Therefore, degrees of freedom were corrected using Greenhouse-Geisser estimates of sphericity ($\varepsilon = .670$). The results revealed a significant effect of time on PsyCap scores, $F(1.340, 118) = 1318.071$, $p < .001$, $\eta^2 = .957$. Post-hoc tests using the Bonferroni correction indicated that PsyCap scores significantly increased from pre-test (M = 37.500, SD = 3.011) to post-test (M = 52.850, SD = 2.130), $p < .001$, and from pre-test to follow-up (M = 50.217, SD = 1.984), $p < .001$. However, the results showed a decrease in PsyCap scores from the post-test to follow-up ($p < .001$) after one week, even though the magnitude of decrease was much smaller than that of the increase during the training. The results provided further evidence that the web-based training significantly improved participants' PsyCap from pre-test to post-test, and yet these effects were gradually fading away over time (Figure 3).

Insert Table 2 Here

Insert Figure 3 Here

PsyCap and outcomes

For **Q3**, we explored how effective this PsyCap training could contribute to sustained teachers' emotional stability in face of daily challenges with ASD students and increase their support after the training. Path analysis was conducted using the treatment group sample from T1 to T3. Specifically, we controled teachers' demographic variables (T1) on PsyCap (T2), and regressed emotional stability (T3) and teacher support (T3) on PsyCap (T2). The proposed path model provided an excellent fit to the data, $\chi^2(8) = 26.995$, CFI = .965, TLI = .955, RMSEA = .006. The results showed that PsyCap after controling teachers' gender, age, education, and tenure was significantly and positively related to emotional stability (β = .051 SE =.005, $p < .001$), and teacher support (β = .069, SE =.005, $p < .001$), indicating that PsyCap developed through the teacher training tended to contribute to higher levels of emotional stability and teacher support one week after completion of the training.

Discussion

Our study implemented a web-based PsyCap training program for teachers in inclusive education. The findings revealed that the 2-hour training was effective in enhancing teachers' PsyCap, including self-efficacy, hope, resilience, and optimism, as evidenced by large effect sizes in the development of these four components during the training. Moreover, while it is reasonable to anticipate a gradual decline in the training effect over time, our follow-up surveys demonstrated that teachers' levels of PsyCap still remained significantly higher compared to their baseline levels before the training. Additionally, the results indicated a positive association between teachers' levels of PsyCap and their emotional stability and support provided to the students in class. These findings were further supported by the responses obtained from the open-ended questions in the follow-up surveys, adding valuable qualitative evidence to scaffold the survey results. Specifically, majority of the

teachers expressed that they gained new knowledge and skills during the training and were impressed by the positive influence of the four components of PsyCap on their well-being and work outcomes. One teacher shared, "What struck me the most was realizing that we have the ability to change things that were once considered unchangeable in our lives. We often tend to be pessimistic about positive changes, but the training showed me the possibility of change... It gives us hope to live positively and effectively cope with challenges in work and life." And most of the teachers agreed that PsyCap is beneficial in the workplace and expressed their intentions to apply the tactics learned from the training in their daily work routines. One teacher mentioned, "During the training, we practiced techniques to enhance our confidence... By breaking down tasks into smaller, manageable steps and successfully completing them, we build confidence in our ability to get things done. For instance, I identified students with learning difficulties as smaller tasks that I must address before tackling larger tasks at work. I created learning profiles for these students to understand them better and provide more effective support when they face setbacks." The teachers also noted that the PsyCap training empowered them to be more supportive in the classroom. They acknowledged that feeling optimistic and hopeful fosters a stronger inclination towards supportive behavior. For example, one teacher reflected, "Every day, we work with many students, some of whom require special attention. It can be challenging to accommodate students with varying developmental levels in a single class, considering their diverse learning abilities and communication skills. The training taught me to cultivate hope by setting goals and breaking them down into manageable steps. I also learned to brainstorm different approaches to accomplish tasks more effectively. I appreciate the brainstorming technique as it provides me with multiple strategies to achieve my goals, which instills a sense of security and calmness. This coping strategy has proven to be very useful, especially

in stressful situations, as regaining control ultimately brings about a sense of calm." To summarize, the teachers highlighted the positive impact of PsyCap on their teaching practices, emphasizing the importance of optimism, confidence, and hope in overcoming challenges and supporting their students. The teachers' feedback demonstrates their appreciation for the PsyCap training and their readiness to embrace positive change for their professional and personal growth.

Contributions and Implications

This study contributed to the inclusive education literature in the following ways. Firstly, it signaled a message that fostering positivity in teachers through trainings might be crucial in challenging situations. Educators and school leaders increasingly recognized the significant impact of teachers' positivity and well-being on their ability to effectively navigate and overcome challenges in different educational contexts (55). By emphasizing the need to foster positivity in teachers through trainings, we assumed that equipping teachers with strategies and skills to cultivate a positive mindset could enhance their resilience and coping abilities when facing various challenges, such as student behavioral issues, workload demands, or limited resources. Furthermore, fostering positivity in teachers not only benefits their own well-being but also has a ripple effect on student outcomes. Teachers who exhibit positive emotions and attitudes are more likely to create a supportive and engaging classroom environment, leading to improved student motivation, learning outcomes, and well-being (56, 57). Past studies indeed highlighted the importance of teacher well-being and positive mindset in promoting student engagement, academic achievement, and a supportive classroom climate (29, 58, 59). Therefore, by equipping teachers with the tools to develop and maintain a positive mindset, we argued that schools could enhance teacher well-being, promote effective classroom management, and ultimately improve student outcomes. To our

best knowledge, few studies have implemented PsyCap training as teacher professional development in Chinese inclusive education contexts. According to Sharma et al. (48), professional development that cultivating positive resources offers a constructive and systematic approach to support teachers in developing a sense of efficacy and competence by equipping them with the necessary knowledge and skills to effectively teach diverse learners. This study emphasized the development of PsyCap through training, which is among the few studies that attempted to nurture hope, optimism, self-efficacy, and resilience in teachers who work with ASD children in the China context.

Moreover, our study contributes to the field by designing and implementing a web-based PsyCap training specifically tailored for inclusive teachers. Web-based PsyCap intervention programs have garnered significant attention in recent years due to their potential in enhancing PsyCap (17, 49, 50). These programs leverage digital platforms to deliver targeted training and support, offering distinct advantages over traditional face-to-face interventions. One major advantage of web-based PsyCap interventions is their accessibility. By utilizing technology, individuals can engage in interventions at their convenience and from various locations. This accessibility expands the reach of interventions, making them available to a larger audience, including those with limited access to face-to-face interventions due to geographical, logistical, or time constraints (60). Another advantage is that web-based interventions are cost-effective compared to face-to-face interventions, as they eliminate the need for physical infrastructure, travel expenses, and instructor fees. This cost-effectiveness enables wider dissemination of interventions and makes them more accessible to organizations and individuals with limited resources (61). By leveraging technology, it becomes feasible to scale up PsyCap training and involve a greater number of teachers in developing their psychological resources to combat workplace stressors and foster

work well-being in challenging situations. In addition, increasing teachers' PsyCap can also affect their awareness of strategies to support ASD students in class. It should be noted that ASD is a highly heterogeneous disability, thus requiring different levels and intensity of support and care from teachers in their day-to-day work (62). Therefore, PsyCap serves as a valuable psychological resource for teachers, protecting them from negative stressors while empowering them to provide tailored support in the inclusive education context.

Limitation and future research directions

The study presented several limitations for future research effort. Firstly, the convenience sampling used in this study may have introduced selection bias, as participants who self-selected for the study might not represent the entire population of teachers. To enhance the generalizability of the findings, future research could employ more robust sampling techniques, such as random sampling or stratified sampling, to ensure a more representative sample of teachers (63). Secondly, the reliance on self-rating measures raises the potential for social desirability bias, as participants may respond in a manner that aligns with societal norms or group expectations. To mitigate this bias, future studies could consider incorporating additional measures, such as observer ratings or behavioral assessments, to provide a more comprehensive and objective evaluation of teachers' experiences and behaviors (64). Furthermore, the one-time-off nature of the PsyCap training in our study limited its long-term impact on teachers' behavior change. Research suggested that sustained and repeated interventions are more effective in producing enduring changes (65). Future studies could explore the effectiveness of extended training programs, including longitudinal interventions or ongoing support, to examine the long-term intervention effects teachers' PsyCap development (66, 67).

Statement and Declarations:

The authors have no conflicts of interest to declare. The authors have seen and agreed with the contents of the manuscript.

The study involving human participants was reviewed and approved by the Department of Special Education and Counselling at The Education University of Hong Kong. Written consent was obtained from the study participants included in the study.

Data is available upon reasonable request from the first author.

Reference

1. Opertti R, Belalcazar C. Trends in Inclusive Education at Regional and Interregional Levels: Issues and Challenges. IBE. 2008.
2. Atta M, Ahmed M, Ghafoor A, Sher H. Implementing inclusive education: Challenges and suggestions. Pakistan Journal of Social Sciences. 2009;29(2):305-313.
3. Maich K, Klinger L, Zoeckler A. Inclusive education: Stories of success and hope in a Canadian context. International Journal of Inclusive Education. 2019;23(8):840-855.
4. Boyle C, Topping K, Jindal-Snape D, Tombs G. The effects of teachers’ beliefs on primary pupils’ beliefs, self-perceptions, and behavior: A longitudinal study. British Journal of Educational Psychology. 2012;82(2):267-280.
5. Domović V, Nikolovska I, Kambovska-Ristovska S. Teachers' attitudes towards inclusive education: A case study from Macedonia. Journal of International Special Needs Education. 2017;20(1):36-46.
6. Tristani L, Bassett-Gunter R. Making the grade: Teacher training for inclusive education: A systematic review. Journal of Research in Special Educational Needs. 2020;20(3):246-264.
7. Cefai C, Cavioni V. Towards inclusion of students with intellectual disability in mainstream classrooms: A review of the literature. International Journal of Inclusive Education. 2014;18(9):949-964.
8. Day C, Sammons P, Hopkins D, Harris A, Leithwood K, Gu Q, et al. The impact of school leadership on pupils' outcomes. Department for Education and Skills. 2007.
9. Forcina V. The effects of teacher stress on learning. ScholarWorks at UMass Boston. 2012.
10. Luthans F, Youssef CM, Avolio BJ. Psychological capital and beyond. Oxford University

Press, USA; 2015.

11. Peterson SJ, Luthans F, Avolio BJ, Walumbwa FO, Zhang Z. Psychological capital and employee performance: A latent growth modeling approach. Personnel Psychology. 2011;64(2):427-450.
12. Luthans F, Youssef CM. Human, social, and now positive psychological capital management: Investing in people for competitive advantage. Organizational Dynamics. 2004;33(2):143-160.
13. Avey JB, Reichard RJ, Luthans F, Mhatre KH. Meta-analysis of the impact of positive psychological capital on employee attitudes, behaviors, and performance. Human resource development quarterly. 2011;22(2):127-152.
14. Cheung SY, Tang CS, Tang KP. Psychological capital as a moderator between emotional labor, burnout, and job satisfaction among school teachers in China. International Journal of Stress Management. 2011;18(4):348-371.
15. Paloș R, Oprea A, Gavreliuc A. Teachers' psychological capital: An examination of its relationship with occupational well-being and work engagement. Frontiers in Psychology. 2020;11:1812.
16. Luthans F, Avey JB, Avolio BJ, Norman SM, Combs GM. Psychological capital development: Toward a micro-intervention. Journal of Organizational Behavior. 2006;27(3):387-393.
17. Luthans F, Avey JB, Patera JL. Experimental analysis of a web-based training intervention to develop positive psychological capital. Academy of Management Learning & Education. 2008;7(2):209-221.
18. Chen H. An exploration of inclusive education in China: Policies, practices, and challenges. In: Forlin C, Loreman T, editors. Measuring inclusive education: International

perspectives. Emerald Publishing Limited; 2017. p. 13-26.

19. McCabe H. Inclusive education in China: Policies, culture and local responses. Comparative Education. 2003;39(2):139-151.

20. Yang L, Zhang D. Inclusive education in China: Perspectives of special education teachers. Frontiers in Education. 2019;4:99.

21. Sun AQ, Xin JF. School principals' opinions about special education services. Preventing School Failure: Alternative Education for Children and Youth. 2020;64(2):106

22. Brackenreed D. Inclusive education: Identifying teachers' strategies for coping with perceived stressors in inclusive classrooms. Canadian Journal of Educational Administration and Policy. 2011; 122.

23. Jennett HK, Harris SL, Mesibov GB. Commitment to philosophy, teacher efficacy, and burnout among teachers of children with autism. Journal of Autism and Developmental Disorders. 2003;33(6):583-593.

24. Aloe AM, Amo LC, Shanahan ME. Classroom management self-efficacy and burnout: A multivariate meta-analysis. Educational Psychology Review. 2014;26:101-126.32.

25. Luthans F. Positive organizational behavior: Developing and managing psychological strengths. Academy of Management Perspectives. 2002;16(1):57-72.

26. Maslow AH. The instinctoid nature of basic needs. Journal of Personality. 1954;22:326–347.

27. Seligman ME, Csikszentmihalyi M. Positive psychology: An introduction. American Psychological Association. 2000;55(1):5.

28. Zhang Y, Zhang S, Hua W. The impact of psychological capital and occupational stress on teacher burnout: Mediating role of coping styles. The Asia-Pacific Education Researcher. 2019;28:339-349.

29. Bakker AB, Demerouti E, Sanz-Vergel AI. Burnout and work engagement: The JD-R approach. Annual Review of Organizational Psychology and Organizational Behavior. 2011;1(1):389-411.

30. Loghman S, Quinn M, Dawkins S, Woods M, Om Sharma S, Scott J. A Comprehensive Meta-Analyses of the Nomological Network of Psychological Capital (PsyCap). Journal of Leadership & Organizational Studies. 2023;30(1):108-128.

31. Chaturvedi SK, Chander R. Construction and validation of the emotional stability scale. Indian Journal of Health and Wellbeing. 2010;1(2):224-227.

32. Guo YF, Wang KF, Cross W, Lam L, Plummer V, Li J. Quality of life in cancer patients with different preferences for nurse spiritual therapeutics: The role of psychological capital. Journal of Advanced Nursing. 2022;78(4):991-1000.

33. Dougall AL, Hyman KB, Hayward MC, McFeeley S, Baum A. Optimism and traumatic stress: The importance of social support and coping. Journal of Applied Social Psychology. 2001;31(2):223-245.

34. Snyder CR, Harris C, Anderson JR, Holleran SA, Irving LM, Sigmon ST, et al. The will and the ways: Development and validation of an individual-differences measure of hope. Journal of Personality and Social Psychology. 2002;60(4):570-585.

35. Bandura A. Self-efficacy: Toward a unifying theory of behavioral change. Psychological Review. 1977;84(2):191-215.

36. Fergus S, Zimmerman MA. Adolescent resilience: A framework for understanding healthy development in the face of risk. Annual Review of Public Health. 2005;26:399-419.

37. Hart D, Donnelly TM, Youniss J, Atkins R. High school community service as a predictor of adult voting and volunteering. American Educational Research Journal. 2013;50(1):5-

29.

38. Koster M, Pijl SJ, Nakken H, Van Houten E. Teachers' perspectives on inclusive education: A systematic review. International Journal of Inclusive Education. 2020;24(8):899-916.

39. Goddard YL, Goddard RD, Kim S. The effects of teacher working conditions on the emotional well-being of teachers and their students' academic achievement. Teaching and Teacher Education. 2021;98:103370.

40. Devereux K, Trachtman RE, Ramos EL, Ravid D. Teacher burnout and its relation to professional development: A systematic review and meta-analysis. Teaching and Teacher Education. 2019;85:1-13.

41. Jia Y, Ying L, Zhou X. Emotional exhaustion and academic achievement: A meta-analysis. Educational Psychology Review. 2019;31(3):539-564.

42. Anderson S, Gallagher T, Moloney R. Inclusive education and social support: Exploring teachers' views. International Journal of Inclusive Education. 2018;22(10):1074-1088.

43. Cobb S. Social support as a moderator of life stress. Psychosomatic Medicine. 1976;38(5):300-314.

44. Tzuriel D, Shamir A. Inclusive education, social support, and student achievement: A multi-level analysis of primary school students. Research in Developmental Disabilities. 2014;35(1):18-27.

45. Lopata C, Thomeer ML, Volker MA. The state of research on teachers' emotional experiences in inclusive classrooms: A review and research agenda. Journal of Autism and Developmental Disorders. 2019;49(9):3393-3406.

46. Borders CM, Bock AM. Preparing teachers for inclusive classrooms: What do preservice teachers need to know? Teacher Education and Special Education. 2012;35(3):193-208.

47. McCrimmon A. Preparing teachers for inclusive classrooms: What aspects of teacher education programs contribute most? Teacher Education Quarterly. 2015;42(3):55-74.
48. Sharma U, Forlin C, Loreman T. Impact of training on pre-service teachers' attitudes and concerns about inclusive education and sentiments about persons with disabilities. Disability & Society. 2008;23(7):773-785.
49. Salanova M, Ortega-Maldonado A. Positive interventions in organizations: Coaching, PsyCap, and flow. In: Bakker AB, editor. Flow at work: Measurement and implications. Routledge; 2019. p. 225-244.
50. Sumalrot S, Pangsap S, Hassenforder E. Psychological capital intervention on tourism workers during the COVID-19 pandemic. Current Issues in Tourism. 2023.
51. Newman A, Ucbasaran D, Zhu FEI, Hirst G. Psychological capital: A review and synthesis. Journal of Organizational Behavior. 2014;35(S1):S120-S138.
52. Luthans F, Avolio BJ, Avey JB, Norman SM. Positive psychological capital: Measurement and relationship with performance and satisfaction. Personnel Psychology. 2007;60(3):541-572.
53. Malecki CK, Demaray MK. Measuring perceived social support: Development of the child and adolescent social support scale (CASSS). Psychology in the Schools. 2002;39(1):1-52.
54. Steel P, Schmidt J, Shultz J. Refining the relationship between personality and subjective well-being. Psychological Bulletin. 2008;134(1):138.
55. Aronson J, Fried CB, Good C. Reducing stereotype threat in classrooms: A review of social-psychological intervention studies on improving the achievement of Black students. Social Issues and Policy Review. 2016;10(1):74-107.
56. Riolli L, Savicki V, Richards J. Psychological capital as a buffer to student stress.

Psychology. 2012;3(12):1164-1171.

57. Wentzel KR. Teacher-student relationships and adolescent competence at school. In: Ladd G, Ryan AL, editors. The handbook of research on schools, schooling, and human development. Routledge; 2016. p. 177-199.
58. Jennings PA, Greenberg MT. The prosocial classroom: Teacher social and emotional competence in relation to student and classroom outcomes. Review of Educational Research. 2009;79(1):491-525.
59. Schaufeli WB, Bakker AB, Salanova M. The measurement of work engagement with a short questionnaire: A cross-national study. Educational and Psychological Measurement. 2008;66(4):701-716.
60. Baumeister RF, Vohs KD, Funder DC. Psychology as the science of self-reports and finger movements: Whatever happened to actual behavior? Perspectives on Psychological Science. 2014;9(3):312-325.
61. Murray E. Web-based interventions for behavior change and self-management: Potential, pitfalls, and progress. Medicine 2.0. 2012;1(2):e3.
62. Ferraioli SJ, Harris SL. Teaching students with autism spectrum disorders: A step-by-step guide for educators. Brookes Publishing. 2011.
63. Johnson RB. Convenience sampling. In: Somekh B, Lewin C, editors. Research methods in the social sciences. SAGE Publications; 2014. p. 103-105.
64. Podsakoff PM, MacKenzie SB, Lee J-Y, Podsakoff NP. Common method biases in behavioral research: A critical review of the literature and recommended remedies. Journal of Applied Psychology. 2003;88(5):879-903.
65. Yousafzai SY, Aboud FE. Review of implementation processes for integrated nutrition and psychosocial stimulation interventions. Annals of the New York Academy of

Sciences. 2014;1308(1):33-45.

66. Hattie J, Masters D, Birch K. Visible learning into action: International case studies of impact. Routledge. 2017.

67. Borman GD, Hewes GM. Long-term effects and cost-effectiveness of elementary school interventions. Journal of Educational Psychology. 2002;94(1):1-8.

Tables

Table 1. Correlations of the key study variables for both the treatment and control groups in post-survey (N =120)

	Mean	SD	Skewness	Kurtosis	1	2	3	4	5	6	7	8	9
1.Age	1.908	.290	-2.866	6.320									
2.Gender	1.744	.767	.476	-1.150	.160								
3.Education	2.542	.721	.126	-.260	-.362**	-.042							
4.Tenure	9.921	8.139	.862	-.482	.805**	.223*	-.388**						
5.Student in Class	30.966	22.897	5.247	31.352	.083	.076	-.119	-.017					
6.Student Age	5.263	1.842	2.711	9.753	-.188*	-.065	.219*	-.170	-.050				
7.Weekly Teaching (min)	151.200	119.884	1.090	.892	.082	-.160	-.153	.101	-.096	.099			
8.PsyCap	51.392	7.701	-.735	-.490	-.080	.092	.131	-.017	-.056	.109	-.022		
9.Emotion Stability	3.999	.598	.017	-1.013	-.068	.123	.158	-.066	.056	.106	-.028	.659**	
10.Teacher Support	4.573	.624	-1.456	.953	-.095	.139	.162	-.050	-.062	.080	-.037	.856**	.615**

Note. *stands for p < .05; ** stands for p < .01 (two-tailed)

Table 2. Correlations of PsyCap from T1 to T2 for treatment group sample (N = 60)

	1	2	3	4	5	6	7	8	9	10	11	12	13	14
1.PsyCapT1														
2.PsyCapT2	.424**													
3.PsyCapT3	.384**	.786**												
4.EfficacyT1	.408**	-.225	-.137											
5.EfficacyT2	.010	.485**	.394**	-.178										
6.EfficacyT3	.043	.288*	.432**	-.042	.752**									
7.HopeT1	.616**	.455**	.352**	.163	.060	.127								
8.HopeT2	.275*	.534**	.490**	-.009	.118	.064	.393**							
9.HopeT3	.306*	.445**	.430**	.009	.006	-.059	.282*	.740**						
10.ResilienceT1	.686**	.526**	.515**	-.011	.217	.175	.268*	.259*	.225					
11.ResilienceT2	.361**	.699**	.506**	-.107	.041	-.141	.394**	.194	.219	.303*				
12.ResilienceT3	.362**	.602**	.619**	-.056	.062	-.068	.308*	.088	.139	.386**	.829**			
13.OptimismT1	.596**	.284*	.178	-.117	-.024	-.100	.187	.085	.148	.284*	.296*	.217		
14.OptimismT2	.345**	.687**	.502**	-.242	.094	.099	.255*	.194	.142	.534**	.353**	.347**	.255*	
15.OptimismT3	.237	.424**	.669**	-.208	.013	-.006	.071	.273*	.249	.437**	.226	.238	.178	.535**

Note. *stands for p < .05; ** stands for p < .01 (two-tailed)

Figure Click here to access/download;Figure;StudyFigures.docx

Figures

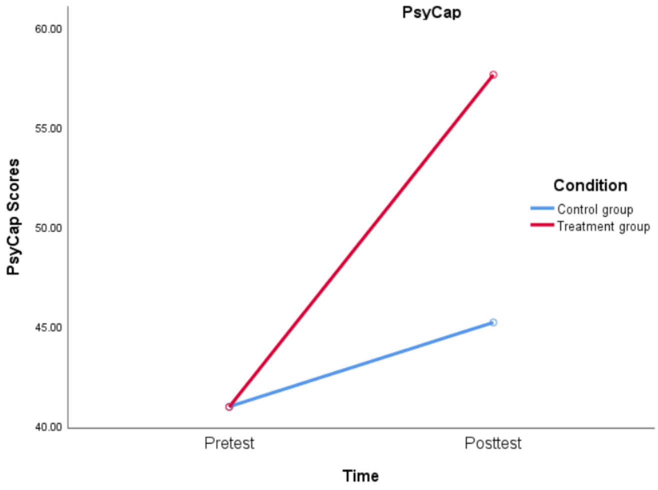

Fig 1. Interaction plot of condition x time on PsyCap scores

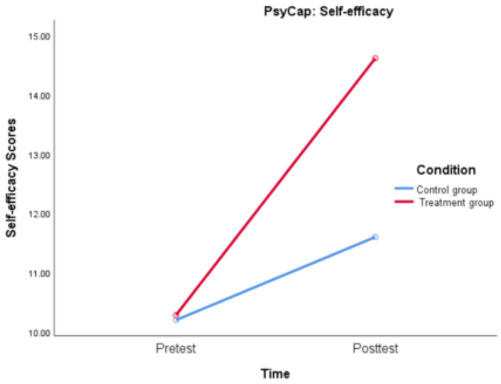

main effect of condition: $F(1, 118) = 75.098$, $p < .001$, $\eta^2 = .389$
main effect of time: $F(1, 118) = 253.111$, $p < .001$, $\eta^2 = .682$
interaction effect: $F(1, 118) = 66.255$, $p < .001$, $\eta^2 = .360$

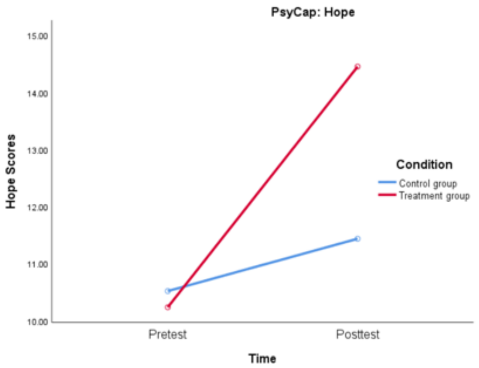

main effect of condition: $F(1, 118) = 36.338$, $p < .001$, $\eta^2 = .235$
main effect of time: $F(1, 118) = 254.339$, $p < .001$, $\eta^2 = .683$
interaction effect: $F(1, 118) = 105.109$, $p < .001$, $\eta^2 = .471$

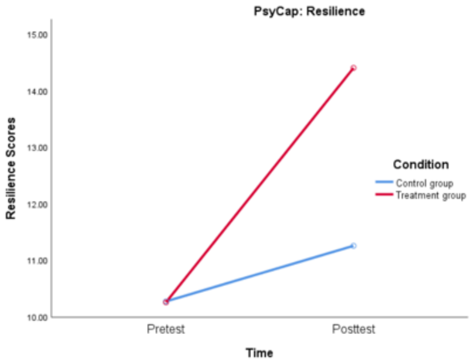

main effect of condition: $F(1, 118) = 37.588$, $p < .001$, $\eta^2 = .242$
main effect of time: $F(1, 118) = 363.487$, $p < .001$, $\eta^2 = .755$
interaction effect: $F(1, 118) = 150.417$, $p < .001$, $\eta^2 = .540$

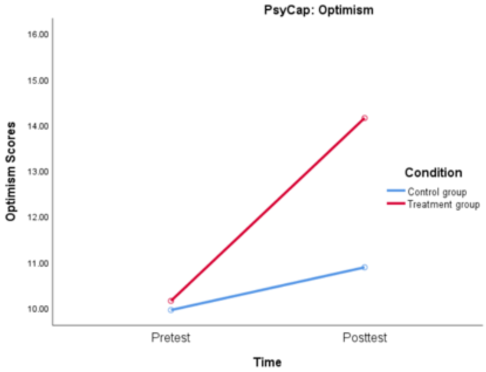

main effect of condition: $F(1, 118) = 70.095$, $p < .001$, $\eta^2 = .373$
main effect of time: $F(1, 118) = 281.800$, $p < .001$, $\eta^2 = .705$
interaction effect: $F(1, 118) = 108.891$, $p < .001$, $\eta^2 = .480$

Fig 2. Interaction plot of condition x time on the scores of the four components of PsyCap

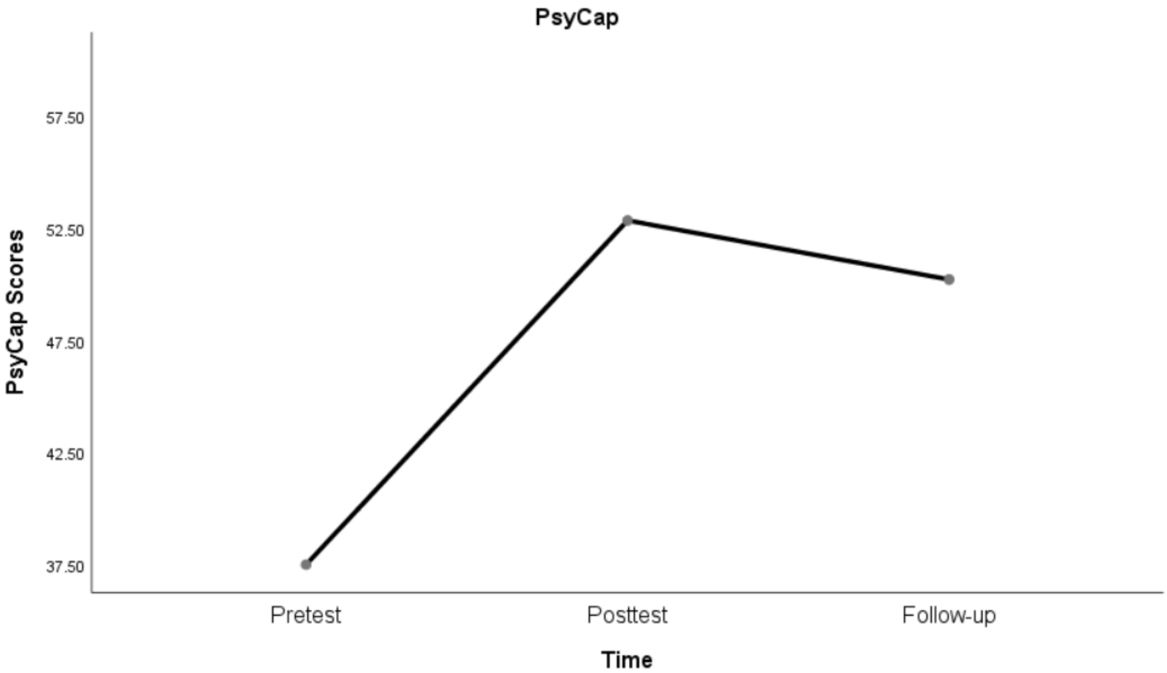

Figure 3. The sustainability of the PsyCap training on teachers' PsyCap scores over time
