# Supplementary material for: Exploring the effects of web-based psychological capitals training on teachers’ PsyCap development, emotional stability, and support: Evidence from Chinese inclusive education
Source: PLoS One. 2024 Sep 30;19(9):e0306453. doi: 10.1371/journal.pone.0306453 (PMC11441704; doi:10.1371/journal.pone.0306453)
Supplement: Attachment — Submitted filename: ResponseLetter.docx [file pone.0306453.s003.docx]

Ref. No.: PONE-D-23-30803

Title: *Exploring the Effects of Web-based Psychological Capitals Training on Teachers' PsyCap Development, Emotional Stability, and Support: Evidence from Chinese Inclusive Education*

Article Type: Full Length Article

Journal Title: PLOS ONE

Corresponding Author: Dr. Yin Ru SHI

All Authors: Dr. Yin Ru SHI; Prof. Kuen Fung Kenneth SIN

Resubmission Date: February 23, 2024

**Comments from Reviewer 1**

**Comment 1:** Thanks for submitting your paper in the PLOS ONE journal. Overall, the present paper is a well-written paper that has a good potential to be accepted for publication. However, before any positive decision, the paper needs to be revised based on the comments included in the manuscript.

**Response 1:** Thank you for your positive feedback. We are grateful for the opportunity to refine our work further. We have carefully considered each point raised in your attachment and have formulated a detailed revision to address them comprehensively. We believe these changes will significantly improve our paper. For your convenience, we have listed your comments one by one with our corresponding responses. Thank you!

**Comment 2:** The format of the current paper should be revised based on the journal's guidelines.

**Response 2:** Thank you for your kind reminder. We have checked the submission guidelines and made adjustments throughout the revised manuscript. Please kindly refer to the revised manuscript for reference.

**Comment 3:** In abstract, the implications of the study should be briefly mentioned here.

**Response 3:** Thank you for your suggestion. We have revised the abstract to make the implications more explicit at its conclusion.

**For your convenience, we have extracted the revised abstract for your reference:**

**“**Inclusive education for students with autism spectrum disorders (ASD) has increasingly received attention nationwide in China. Schools realize that teachers are under stress and lack the skills to handle daily interactions with these students. So far, few studies have directed efforts to provide a remedy for teachers to improve their daily work. This study aimed to design and implement a 2-hour web-based training on psychological capital (PsyCap) to protect their well-being and foster their supportive behavior for ASD students in the inclusive class. A total of 120 targeted teachers were invited to participate in the training and were randomly divided into control and treatment groups. Pre-, post-, and follow-up surveys were distributed before, after, and one week after the training. ANOVA results suggested that teachers showed a significant increase in their PsyCap scores after completing the training, although the training effect slightly decreased after one week. Moreover, higher levels of PsyCap showed a positive influence on teachers' emotional stability and supportive behavior in class. **The results highlighted the effectiveness of the web-based PsyCap training in boosting teacher positivity, which enhances teacher support for students in inclusive education.**”

**Comment 4:** Where is the introduction? Prior to explaining the variables and reviewing the pertinent literature you need to talk about the existing gaps, research aims, and significance of the study.

**Response 4:** Thank you for your valuable reminder regarding our manuscript structure. Upon reflection, we recognize that the initial presentation of the introduction, which encompassed within the first two paragraphs and delineating the three research questions, may not have conformed precisely to the journal's formatting guidelines. This oversight likely contributed to the lack of clarity regarding the section's designation.

To adhere to the highest standards of clarity and coherence, we have taken immediate steps to rectify this issue. Specifically, we have revised the manuscript to include a distinct section title for the introduction, in strict accordance with the journal's formatting requirements.

In addition, we appreciate your insights highlighting the necessity for a clear introduction that outlines the existing research gaps, states the aims of the study, and emphasizes its significance within the broader field. In response to your feedback, we have further refined the introduction section in our revised manuscript.

**For your convenience, we have extracted the revised introduction for your reference:**

**“Introduction**

Inclusive education receives a growing concern from international communities as increasingly more educational reforms have taken place in both developing and developed regions for a heightened understanding of education as a human right (1). Despite this growing concern, the practical implementation of inclusive education encounters significant challenges, particularly in accommodating the varied developmental, cultural, socioeconomic, and familial backgrounds of students, including those with Autism Spectrum Disorders (ASD) (2). Among these diverse student populations, children with ASD stand out as a minority group that demands considerable attention due to the complexities inherent in meeting their educational and social needs (3). In this context, teachers emerge as critical agents for the successful enactment of inclusive practices, tasked with the dual responsibility of facilitating an inclusive classroom environment while addressing the special needs of each student (4,5).

However, a notable gap in the literature reveals that teachers frequently feel not adequately equipped for this role, particularly in relation to effectively integrating children with ASD into their classrooms (6). This gap is exacerbated by the pressures of educational reforms, which impose heightened standards and accountability, further compounded by challenges such as student misconduct and the demand for individualized care (7, 8). These factors collectively contribute to increased stress, pessimism, and a decline in motivation among teachers, negatively impacting their overall wellbeing and job satisfaction. In the quest for solutions to these challenges, the concept of Psychological Capitals (PsyCap) emerges as a promising avenue for research and practice (10). PsyCap, with its components of hope, optimism, resilience, and self-efficacy, is posited as the vital psychological resources for enhancing individual performance, cognitive functioning, and motivation towards success (11, 12). Despite the established benefits of PsyCap in various organizational settings, its application within the context of inclusive education, particularly as a support for teachers facing the multifaceted challenges of integrating ASD students, remains underexplored.

This study aims to bridge this critical gap by examining the potential of PsyCap to empower teachers within inclusive educational settings. Specifically, it seeks to design and evaluate a PsyCap training intervention in enhancing teachers' psychological resources, thereby facilitating a more positive and effective teaching environment for students with ASD. The significance of this research lies in its potential to offer evidence-based strategies for improving teacher preparedness and resilience, contributing to the broader goals of inclusive education by ensuring that teachers are well-equipped to meet the diverse needs of all students (5, 9). Past studies underscore the transformative potential of PsyCap, with studies indicating its negative correlation with undesirable employee attitudes and behaviors, and a positive association with beneficial outcomes such as job satisfaction, performance, and well-being (13, 14, 15).

By focusing on the development and implementation of an online PsyCap training program, inspired by the pioneering work of Luthans and his associates (16, 17), this study tries to showcase how the PsyCap training can bolster the psychological resources of teachers. Prior investigations have demonstrated the feasibility and effectiveness of developing PsyCap through brief, technology-facilitated interventions (17). For instance, Luthans et al. (17) illustrated that an intensive 2-hour web-based training could significantly enhance university students' PsyCap, leading to notable improvements in self-efficacy, optimism, and hope. Drawing upon these findings, the current study proposes a similar online training tailored for teachers in inclusive settings, aiming to equip them with the necessary psychological tools to address the challenges and stressors inherent in their roles. Building upon these foundations, this study not only addresses the existing research gap but also proposes a practical intervention that can potentially enhance the inclusivity and effectiveness of educational practices, thereby supporting the professional development of teachers and the academic success of students with ASD. This study answers the following research questions:

RQ1: Can teachers’ PsyCap be effectively developed through the short web-

based PsyCap training?

RQ2: Can this PsyCap training show sustained positive effects on teachers’
 PsyCap to be developed?

RQ3: How effective can this PsyCap training on teachers’ sustained well-being

and support to students?**”**

**Comment 5:** Prior to describing the data collection procedure, you need to explain the setting and participants.

**Response 5:** Thank you for your comment. We have added this new section accordingly.

**For your convenience, we have extracted the new content for your reference:**

“**Sample participants**

Recruitment of the target teachers started on April 20th, 2020. In this study, 178 teachers teaching inclusive classes in a coastal city of China were invited. Target teachers were contacted through personal email for participation consent in the online “Positive Quality Teacher Training” project. All the participants provided informed consent after receiving a comprehensive explanation of the research aims and procedure details, emphasizing their right to withdraw at any time without consequences. They were ensured privacy and confidentiality through stringent anonymization and secure storage practices. In this study, the PsyCap training was designed to minimize any potential harm to participants, ensuring they experienced no physical, psychological, or emotional distress. In total, 120 of them agreed to participate and included for data analysis (response rate = .67). Most of the teachers were female (90.1%). Their age ranged from 21 to 50, with 47.6% between 21 and 30, 32.2% between 31 and 40, and 2.1 percent missing. Most of them (84.7%) received a university education. Their work tenure ranged from 1 year to 30 years (Mean = 15.55, SD = 8.67). The average age of the students they taught was 8.6 years old (SD = 2.31).”

**Comment 6:** As you know, in the first paragraph of discussion, the aims and objectives of the study need to be briefly mentioned.

**Response 6:** Thank you for your constructive comment. In response to your valuable feedback, we have revised the first paragraph of the discussion section to succinctly reiterate the study’s aims and objectives.

**For your convenience, we have extracted the new content in revised discussion for your reference:**

“**Discussion**

**The primary aim of this study was to investigate the effectiveness of a web-based PsyCap training program tailored specifically for teachers engaged in inclusive education. The study objectives were to determine whether such a program could enhance key components of PsyCap—self-efficacy, hope, resilience, and optimism—and to establish the sustainability of these enhancements over time. Furthermore, this study sought to explore the potential correlation between increased PsyCap among teachers and the quality of support provided to students, particularly those with additional educational needs.**

The findings revealed that the 2-hour training was effective in enhancing teachers' PsyCap, including self-efficacy, hope, resilience, and optimism, as evidenced by large effect sizes in the development of these four components during the training. Such positive outcome resonates with the theoretical underpinnings of PsyCap posited by Luthans (25), suggesting that such psychological resources are crucial for performance improvement and stress reduction in the workplace. The sustainability of the training effects, evidenced by the maintained levels of PsyCap among teachers over time, addresses a critical gap highlighted in previous research (20, 23). It suggests that the web-based training not only facilitates the initial development of these psychological capitals but also supports their sustainability, countering the traditional concerns regarding the ephemeral nature of training outcomes. This enduring effect is particularly noteworthy given the challenges of implementing inclusive education in China, such as insufficient resources and teacher preparation (18, 20). Moreover, the observed positive correlation between enhanced PsyCap levels and improved emotional stability and support for students aligns with findings from Jennett et al. (23), who warned of the heightened risk of burnout among teachers in inclusive settings. This study contributes to the literature by demonstrating that enhancing teachers' psychological resources can mitigate such risks, fostering a more supportive and effective educational environment for students with additional needs.

The results of the study lend empirical support to the advocacy for cultivating PsyCap among teachers (25, 26), suggesting that such interventions can indeed lead to increased job satisfaction, reduced stress and burnout, and improved student outcomes (28). The study findings were further supported by the teachers’ feedback obtained from the open-ended questions in the follow-up surveys, adding valuable qualitative evidence to scaffold the survey results. The feedback from teachers indicates the practical applicability and transformative potential of PsyCap training. This aligns with the broader educational goals of increasing school enrolment and retention by integrating children with ASD and other special needs into mainstream schools (19). The majority highlighted the acquisition of new knowledge and skills for their well-being and work outcomes. One teacher mentioned, "What struck me the most was realizing that we have the ability to change things that were once considered unchangeable in our lives... It gives us hope to live positively and effectively cope with challenges in work and life." This sentiment reflects a shared recognition among teachers of PsyCap's benefits in fostering a positive workplace environment and enhancing their teaching practices. Some teachers also detailed how the training equipped them with strategies to boost confidence and manage classroom challenges more effectively. "During the training, we practiced techniques to enhance our confidence... I created learning profiles for these students to understand them better and provide more effective support when they face setbacks." Moreover, the training inspired a more supportive attitude in the classroom, with teachers acknowledging the role of optimism and hope in promoting supportive behavior towards students. "The training taught me to cultivate hope by setting goals and breaking them down into manageable steps... This coping strategy has proven to be very useful, especially in stressful situations, as regaining control ultimately brings about a sense of calm," reflected a teacher, emphasizing the utility of PsyCap in navigating the complexities of inclusive education.”

**Comment 7:** From line 514-537, too long paragraph. Please break it into two or more paragraphs.

**Response 7:** We appreciate your observation regarding the lengthy paragraph spanning lines 514-537 and acknowledge the importance of readability and clarity in our manuscript. In response, we have revised this section by dividing it into smaller, more digestible paragraphs in the revised manuscript. In fact, we have made adjustments regarding this issue throughout the manuscript. Please kindly refer to the revised manuscript for such updates.

**Comments from Reviewer 2**

**Comment 1:** Thank you very much for offering me this valuable chance to review this illuminating article entitled “Exploring the Effects of Web-based Psychological Capitals Training on Teachers'PsyCap Development, Emotional Stability, and Support: Evidence from Chinese Inclusive Education”. The study clearly touches the theoretical and empirical bases of the variables separately and connects them logically. It truly adds to the current literature regarding students with autism spectrum disorders (ASD). Despite these merits, the study can improve by considering the following minor revisions. In case the next version is amended, the manuscript can be accepted.

**Response 1:** We are grateful for the opportunity to receive your insights and for the positive remarks about how our study contributes to the existing literature on students with ASD.

We acknowledge the importance of your suggestions for revisions to further enhance the quality and impact of our work. We have committed to addressing these recommendations thoroughly and have incorporated the necessary amendments into the revised manuscript. In the following, we tried to address your suggestions and comments one by one.

**Comment 2:** In the abstract, revise the last sentence “Our findings highlighted the importance of nurturing teachers' PsyCap…..” Use the results highlighted……..

**Response 2:** We thank you for your feedback concerning the use of first-person terminology in our manuscript. In response to your valuable suggestion, we have thoroughly revised the manuscript to replace first-person phrases like "our finding", “Our study”, and “we consider” with third-person expressions. We have ensured that terms, such as "the findings of this study", "the results indicate", “This study adopted”, or “xxx was examined”, are used to maintain an objective and professional tone. Please kindly refer to the revised manuscript for this update. Thank you!

**For your convenience, we have extracted the revised abstract for your reference:**

“Inclusive education for students with autism spectrum disorders (ASD) has increasingly received attention nationwide in China. Schools realize that teachers are under stress and lack the skills to handle daily interactions with these students. So far, few studies have directed efforts to provide a remedy for teachers to improve their daily work. This study aimed to design and implement a 2-hour web-based training on psychological capital (PsyCap) to protect their well-being and foster their supportive behavior for ASD students in the inclusive class. A total of 120 targeted teachers were invited to participate in the training and were randomly divided into control and treatment groups. Pre-, post-, and follow-up surveys were distributed before, after, and one week after the training. ANOVA results suggested that teachers showed a significant increase in their PsyCap scores after completing the training, although the training effect slightly decreased after one week. Moreover, higher levels of PsyCap showed a positive influence on teachers' emotional stability and supportive behavior in class. The results highlighted the effectiveness of the web-based PsyCap training in boosting teacher positivity, which enhances teacher support for students in inclusive education.”

**Comment 3:** Add an introduction section to the paper (3-4 paragraphs). The current way of presenting information resembles LR. You need to follow “Introduction, LR, Method, Results, Discussion, Conclusion sections.

**Response 3:** Thank you for your comment. In fact, the first two paragraphs in the original manuscript were Introduction. However, we realized that the initial presentation of the introduction, contained within the first two paragraphs and outlining the three research questions, may not have fully adhered to the journal's formatting guidelines. This discrepancy likely led to confusion of “missing Introduction”.

To ensure clarity in manuscript structure, corrective actions have been undertaken. The manuscript has now been updated to feature a clearly defined section title for the introduction, aligning precisely with the journal's formatting standards.

Apart from this, we have further consolidated and polished the introduction section in the revised manuscript highlighting the necessity for a clear introduction that outlines (1) the existing research gap, (2) states the aims of the study, and (3) emphasizes its significance within the broader field.

**For your convenience, we have extracted the revised introduction for your reference:**

**“Introduction**

Inclusive education receives a growing concern from international communities as increasingly more educational reforms have taken place in both developing and developed regions for a heightened understanding of education as a human right (1). Despite this growing concern, the practical implementation of inclusive education encounters significant challenges, particularly in accommodating the varied developmental, cultural, socioeconomic, and familial backgrounds of students, including those with Autism Spectrum Disorders (ASD) (2). Among these diverse student populations, children with ASD stand out as a minority group that demands considerable attention due to the complexities inherent in meeting their educational and social needs (3). In this context, teachers emerge as critical agents for the successful enactment of inclusive practices, tasked with the dual responsibility of facilitating an inclusive classroom environment while addressing the special needs of each student (4,5).

However, a notable gap in the literature reveals that teachers frequently feel not adequately equipped for this role, particularly in relation to effectively integrating children with ASD into their classrooms (6). This gap is exacerbated by the pressures of educational reforms, which impose heightened standards and accountability, further compounded by challenges such as student misconduct and the demand for individualized care (7, 8). These factors collectively contribute to increased stress, pessimism, and a decline in motivation among teachers, negatively impacting their overall wellbeing and job satisfaction. In the quest for solutions to these challenges, the concept of Psychological Capitals (PsyCap) emerges as a promising avenue for research and practice (10). PsyCap, with its components of hope, optimism, resilience, and self-efficacy, is posited as the vital psychological resources for enhancing individual performance, cognitive functioning, and motivation towards success (11, 12). Despite the established benefits of PsyCap in various organizational settings, its application within the context of inclusive education, particularly as a support for teachers facing the multifaceted challenges of integrating ASD students, remains underexplored.

This study aims to bridge this critical gap by examining the potential of PsyCap to empower teachers within inclusive educational settings. Specifically, it seeks to design and evaluate a PsyCap training intervention in enhancing teachers' psychological resources, thereby facilitating a more positive and effective teaching environment for students with ASD. The significance of this research lies in its potential to offer evidence-based strategies for improving teacher preparedness and resilience, contributing to the broader goals of inclusive education by ensuring that teachers are well-equipped to meet the diverse needs of all students (5, 9). Past studies underscore the transformative potential of PsyCap, with studies indicating its negative correlation with undesirable employee attitudes and behaviors, and a positive association with beneficial outcomes such as job satisfaction, performance, and well-being (13, 14, 15).

By focusing on the development and implementation of an online PsyCap training program, inspired by the pioneering work of Luthans and his associates (16, 17), this study tries to showcase how the PsyCap training can bolster the psychological resources of teachers. Prior investigations have demonstrated the feasibility and effectiveness of developing PsyCap through brief, technology-facilitated interventions (17). For instance, Luthans et al. (17) illustrated that an intensive 2-hour web-based training could significantly enhance university students' PsyCap, leading to notable improvements in self-efficacy, optimism, and hope. Drawing upon these findings, the current study proposes a similar online training tailored for teachers in inclusive settings, aiming to equip them with the necessary psychological tools to address the challenges and stressors inherent in their roles. Building upon these foundations, this study not only addresses the existing research gap but also proposes a practical intervention that can potentially enhance the inclusivity and effectiveness of educational practices, thereby supporting the professional development of teachers and the academic success of students with ASD. This study answers the following research questions:

RQ1: Can teachers’ PsyCap be effectively developed through the short web-

based PsyCap training?

RQ2: Can this PsyCap training show sustained positive effects on teachers’

PsyCap to be developed?

RQ3: How effective can this PsyCap training on teachers’ sustained well-being

and support to students?”

**Comment 4:** The gaps can be further explained. Why is it significant?

**Response 4:** Thank you for your comment. Please kindly refer to our response to your comment 3. We have revised the introduction section with a clearer emphasis on the research gap, the study aim, and the significance of the study.

**Comment 5:** You can add a figure to “PsyCap with Four Components”.

**Response 5:** Thank you for your suggestion. We have added a figure depicting the relationship between PsyCap and its four components to our revised manuscript.

**For your convenience, we have extracted the new figure for your reference:**

**Fig.** PsyCap and its four components

**Comment 6:** Some paragraphs are too long. Please, break them into shorter ones.

**Response 6:** Your observation regarding the lengthy paragraph is appreciated, and the importance of readability and clarity in the manuscript is acknowledged. In response, we further have consolidated and refined the manuscript. The revised manuscript now has been revised into smaller, more digestible paragraphs. For this update, please kindly refer to the revised manuscript.

**Comment 7:** In the participants section, please add mean and SD of the participants’ age range and other background information. The readers need to know some demographics for replication.

**Response 7:** Thank you very much for your thoughtful suggestion. We value your input and would like to assure you that we include all available demographic information in this section. For example, we have added mean and standard deviation for teacher tenure. However, for some other background information, we were not able to include as they were not collected during the course of this study.

We also gratefully acknowledge your recommendation to include standard deviation and mean values for age. However, in our study's design, age was treated as a categorical variable, with participants selecting from a range of age categories. As such, the calculation of mean and standard deviation would not be applicable to the format of our data collection. We hope this clarifies our methodology and thank you once again for your engagement with our work.

**Please indicate your age range by checking the box.**

**“21-30,**

**31-40,**

**41-50,**

**51-60,**

**60+”**

**Comment 8:** Explain why you have chosen this design.

**Response 8:** Thank you for the comment. We have added explanations why we used the quasi-experimental design for measuring the effectiveness of our PsyCap training in our revised manuscript.

**For your convenience, we have extracted the revised section for your reference:**

“**Data collection**

This study used a pre- and post-test quasi-experimental design. **This design was chosen primarily for its strength in evaluating the effects of an intervention when random assignment might not be feasible due to logistical considerations. It allows us to observe the changes in the dependent variable before and after the intervention in the same group of subjects, thereby providing a clearer picture of the intervention’s impact.** The sample size for the treatment group included 60 target teachers, and the control group contained 60 teachers. The teachers were randomly assigned into the two groups. Teachers in the treatment group were provided with the online PsyCap Training, while teachers in the control group were not given the training, but had a decision-making exercise instead.

The online pre- and post-surveys were developed in a Chinese data collection platform. The target teachers were asked to create their own unique ID with a combination of their surname and the last four digits of their phone number, such that their pre- and post-surveys could be matched after submission. These teachers completed their demographic information and baseline PsyCap (T1) one week before they were randomly assigned to either a 2-hour PsyCap teacher training or a decision-making exercise. Both treatment and control groups received a post-survey on PsyCap, emotional stability, and teacher support upon completion of the intervention (T2). After a week, the teachers in the treatment group were further invited for a follow-up survey (T3) on these key variables.”

**Comment 9:** How did you ensure ethics of research? Which ethics did you observe in the study? Add them please.

**Response 9:** Thank you for emphasizing the importance of addressing the ethical considerations involved in our study. We recognize the omission of explicitly stated ethical measures and oversight in our initial manuscript and appreciate the opportunity to address this crucial aspect in our revised method section.

In response to your feedback, we have revised our manuscript to include a comprehensive 'ethical considerations' paragraph in the method section. We hope this amendment can enhance the transparency and ethical rigor of our work. Thank you!

**For your convenience, we have extracted the new content in method for your reference:**

“**Sample participants**

Recruitment of the target teachers started on April 20th, 2020. In this study, 178 teachers teaching inclusive classes in a coastal city of China were invited. **Target teachers were contacted through personal email for participation consent in the online “Positive Quality Teacher Training” project.** **All the participants provided informed consent after receiving a comprehensive explanation of the research aims and procedure details, emphasizing their right to withdraw at any time without consequences. They were ensured privacy and confidentiality through stringent anonymization and secure storage practices. In this study, the PsyCap training was designed to minimize any potential harm to participants, ensuring they experienced no physical, psychological, or emotional distress.** In total, 120 of them agreed to participate and included for data analysis (response rate = .67). Most of the teachers were female (90.1%). Their age ranged from 21 to 50, with 47.6% between 21 and 30, 32.2% between 31 and 40, and 2.1 percent missing. Most of them (84.7%) received a university education. Their work tenure ranged from 1 year to 30 years (Mean = 15.55, SD = 8.67). The average age of the students they taught was 8.6 years old (SD = 2.31).”

**Comment 10:** In discussing your findings, try to connect to theoretical foundations mentioned in LR.

**Response 10:** Thank you for your constructive feedback. We appreciate your suggestion to more closely align the discussion of our findings with the theoretical foundations outlined in the Literature Review. In the revised manuscript, we have ensured to draw clearer connections between our findings and the studies discussed in the Literature Review, particularly focusing on how our results support, extend, or challenge these existing studies.

**For your convenience, we have extracted the new content in revised discussion for your reference:**

“**Discussion**

The primary aim of this study was to investigate the effectiveness of a web-based PsyCap training program tailored specifically for teachers engaged in inclusive education. The study objectives were to determine whether such a program could enhance key components of PsyCap—self-efficacy, hope, resilience, and optimism—and to establish the sustainability of these enhancements over time. Furthermore, this study sought to explore the potential correlation between increased PsyCap among teachers and the quality of support provided to students, particularly those with additional educational needs.

The results revealed that the 2-hour training was effective in enhancing teachers' PsyCap, including self-efficacy, hope, resilience, and optimism, as evidenced by large effect sizes in the development of these four components during the training. Such positive outcome resonates with the theoretical underpinnings of PsyCap posited by Luthans (25), suggesting that such psychological resources are crucial for performance improvement and stress reduction in the workplace. The sustainability of the training effects, evidenced by the maintained levels of PsyCap among teachers over time, addresses a critical gap highlighted in previous research (20, 23). It suggests that the web-based training not only facilitates the initial development of these psychological capitals but also supports their sustainability, countering the traditional concerns regarding the ephemeral nature of training outcomes. This enduring effect is particularly noteworthy given the challenges of implementing inclusive education in China, such as insufficient resources and teacher preparation (18, 20). Moreover, the observed positive correlation between enhanced PsyCap levels and improved emotional stability and support for students aligns with findings from Jennett et al. (23), who warned of the heightened risk of burnout among teachers in inclusive settings. This study contributes to the literature by demonstrating that enhancing teachers' psychological resources can mitigate such risks, fostering a more supportive and effective educational environment for students with additional needs.

The results of the study lend empirical support to the advocacy for cultivating PsyCap among teachers (25, 26), suggesting that such interventions can indeed lead to increased job satisfaction, reduced stress and burnout, and improved student outcomes (28). The study findings were further supported by the teachers’ feedback obtained from the open-ended questions in the follow-up surveys, adding valuable qualitative evidence to scaffold the survey results. The feedback from teachers indicates the practical applicability and transformative potential of PsyCap training. This aligns with the broader educational goals of increasing school enrolment and retention by integrating children with ASD and other special needs into mainstream schools (19). The majority highlighted the acquisition of new knowledge and skills for their well-being and work outcomes. One teacher mentioned, "What struck me the most was realizing that we have the ability to change things that were once considered unchangeable in our lives... It gives us hope to live positively and effectively cope with challenges in work and life." This sentiment reflects a shared recognition among teachers of PsyCap's benefits in fostering a positive workplace environment and enhancing their teaching practices. Some teachers also detailed how the training equipped them with strategies to boost confidence and manage classroom challenges more effectively. "During the training, we practiced techniques to enhance our confidence... I created learning profiles for these students to understand them better and provide more effective support when they face setbacks." Moreover, the training inspired a more supportive attitude in the classroom, with teachers acknowledging the role of optimism and hope in promoting supportive behavior towards students. "The training taught me to cultivate hope by setting goals and breaking them down into manageable steps... This coping strategy has proven to be very useful, especially in stressful situations, as regaining control ultimately brings about a sense of calm," reflected a teacher, emphasizing the utility of PsyCap in navigating the complexities of inclusive education.”
